# Protective effects of gelsolin in acute pulmonary thromboembolism and thrombosis in the carotid artery of mice

**DOI:** 10.1371/journal.pone.0215717

**Published:** 2019-04-19

**Authors:** Ashok Kumar Gupta, Bhupinder Singh Chopra, Bhavna Vaid, Amin Sagar, Sachin Raut, Maulik D. Badmalia, Ashish, Neeraj Khatri

**Affiliations:** 1 Division of Animal Facility, CSIR - Institute of Microbial Technology, Chandigarh, India; 2 Division of Protein Science & Engineering, CSIR - Institute of Microbial Technology, Chandigarh, India; Maastricht University Medical Center, NETHERLANDS

## Abstract

The present study provides first evidence on the role of plasma gelsolin in protecting pulmonary thromboembolism and thrombosis in a mouse model. Gelsolin is the most abundant actin depolymerizing protein in plasma and its significantly depleted values have been reported in metabolic disorders including cardiovascular diseases and myocardial infarction. Though gelsolin replacement therapy (GRT) has been shown to be effective in some animal models, no such study has been reported for thrombotic diseases that are acutely in need of bio-therapeutics for immediate and lasting relief. Here, using mice model and recombinant human gelsolin (rhuGSN), we demonstrate the antithrombotic effect of gelsolin in ferric chloride induced thrombosis in carotid artery and thrombin induced acute pulmonary thromboembolism. In thrombosis model, arterial occlusion time was significantly enhanced upon subcutaneous (SC) treatment with 8 mg of gelsolin per mice viz. 15.83 minutes vs. 8 minutes in the placebo group. Pertinently, histopathological examination showed channel formation within the thrombi in the carotid artery following injection of gelsolin. Fluorescence molecular tomography imaging further confirmed that administration of gelsolin reduced thrombus formation following carotid artery injury. In thrombin-induced acute pulmonary thromboembolism, mice pretreated with aspirin or gelsolin showed 100 and 83.33% recovery, respectively. In contrast, complete mortality of mice was observed in vehicle treated group within 5 minutes of thrombin injection. Overall, our studies provide conclusive evidence on the thrombo-protective role of plasma gelsolin in mice model of pulmonary thromboembolism and thrombosis.

## Introduction

Globally, cardiovascular and cerebrovascular diseases as well as venous thromboembolism are the cause of 17 million deaths annually[1] and are projected to be the top two major causes of death by 2030[2]. Considering the paucity of FDA-approved biotherapeutics for short- and long-term interventions, the cost of medical care for these conditions is immense and is increasing every year. Platelets play a crucial role in the pathogenesis of thrombosis[3, 4], essentially arterial and microvascular thrombi that in turn is accountable for vascular diseases[5, 6]. Presently, pharmacological approaches to treat these thromboembolic disorders focus on therapies utilizing anticoagulants such as heparin[7]. Blood viscosity is known to increase in these cases due to “spillage” of F-actin in plasma due to local tissue injury and/or cell death[8]. This further aggravates the condition by causing occlusions and sharply reduces the availability of natural anti-clotting agents.

Interestingly, plasma form of gelsolin (pGSN) is an abundant actin regulating protein that depolymerizes filamentous actin released in circulation following tissue injury/cell death and remains attached to depolymerized end by capping it. GSN exists in three forms: intracellular or cytoplasmic, plasma and periplasmic. Plasma gelsolin (pGSN) is an 85.7 kDa protein and uses a large pool of free calcium in plasma (~1 mM) for its actin depolymerization and capping activity[9]. A major proportion of pGSN is secreted by smooth, skeletal and cardiac muscle cells[10]. Gelsolin is also expressed in platelets and plays a pivotal role in cytoskeletal reorganization[11]. The cytoskeleton is primarily composed of actin in globular (G-actin) as well as polymerized (F-actin) forms[11, 12]. The circulating concentration of pGSN in human plasma varies from 200–300 mg/L[13–15] and has been shown to exhibit a half-life of 2–3 days in humans and rabbits[16]. Decreased levels of pGSN have also been previously reported in animal models of Alzheimer’s disease[17], hyperoxia[18], oleic acid induced lung injury[19], cecal ligation/puncture model of sepsis, lipopolysaccharide (LPS, endotoxin) challenge[20, 21], stroke[22] and diabetes[23]. Earlier studies have reported beneficial effects of gelsolin replacement therapy (GRT) in animal model of diabetes[23], sepsis, burn[24] and inflammation[25]. pGSN levels have been reported to decline by 20–50% in multiple human diseases such as sepsis, major trauma, multiple organ dysfunction syndromes (MODS), rheumatoid arthritis, haemodialysis, multiple sclerosis, Alzheimer’s disease, tick-borne encephalitis and lymeborreliosis[17], acute liver injury and myocardial infarction[26], stroke[27], malaria[28], allogenic stem cell transplantation[29]. In stroke patients also, plasma gelsolin (pGSN) levels have been reported to decline significantly and a negative correlation with pGSN level was observed with the National Institute of Health Stroke Score/Scale (NIHSS) and C-reactive protein levels[27]. Considering the therapeutic potential of GRT, GRT is in Phase 2 trials for community acquired pneumonia (CAP), trauma and sepsis (NCT03466073). Le *et al*. have previously reported therapeutic effects of administration of pGSN in transient focal ischemia in rats[30]. Given that pGSN plays a critical role in recovery from injuries and cytoskeleton reorganization of platelets, the current study is directed to evaluate the protective role of GRT in carotid artery thrombosis and pulmonary thromboembolism in mice.

## Materials and methods

### Ethics statement

The Animal Ethics Committee of CSIR-IMTECH, Chandigarh (Approval number IAEC/13/23), approved the experimental protocols.

### Chemicals, drugs and reagents

Recombinant human gelsolin (rhuGSN) was expressed and purified as described previously[31]. As used in other studies, high (96%) sequence similarity between human and mice pGSN allowed us to use rhuGSN for experimentation in mice[31]. Thrombin (human plasma) (Calbiochem, USA), Aspirin (Sigma, China), Ferric chloride (Sigma, Germany) and Whatman filter paper (GE Healthcare, UK) were used in this study. AngioSense 750EX for fluorescence molecular tomography imaging was purchased from PerkinElmer Life Sciences, Waltham, MA.

### Animals

Female BALB/c mice (20–25 g, 8 weeks old) used in this study were obtained from the Animal Facility of the Institute of Microbial Technology (IMTECH). Mice were housed in a state-of-the-art barrier maintained Animal Facility in individually ventilated cages and were provided feed and water *ad libitum*. The experimental protocols were approved by the Animal Ethics Committee of IMTECH, Chandigarh (approval number IAEC/13/23) and performed as per the principles and guidelines of the Committee for the Purpose of Supervision of Experiments on Animals (CPCSEA), Ministry of Environment, Forests & Climate Change, India. All efforts were undertaken to minimize pain, suffering, and distress in animals by using analgesics or anaesthetics as and when required. Mice were monitored throughout the experiment period and all experimental protocols on animals were performed by the trained research staff.

### Ferric chloride induced arterial thrombosis

A mouse model of ferric chloride induced carotid artery thrombosis was used as described earlier[32]. Mice (n = 6) of different groups were pretreated either with vehicle or rhuGSN (8 mg/mouse, subcutaneously, SC) 30 minutes prior to the application of ferric chloride solution. Mice were anesthetized by intraperitoneal cocktail injection of ketamine (100mg/kg) and xylazine (10mg/kg). Left common carotid artery was surgically exposed and a Laser Doppler Flow (LDF) probe (AD Instruments) was placed on the surface of the artery. As described before, Whatman filter paper no. 1 (1 x 2 mm) saturated with 10% ferric chloride was placed on the surface of the carotid artery[33]. After 3 minutes, the filter paper was removed from the artery surface and saline solution was used to wipe the excess ferric chloride. Blood flow in the carotid artery of vehicle and rhuGSN treated groups was monitored using LDF. The time required for thrombotic occlusion after initiation of arterial injury by applying the filter paper was monitored.

#### Percent blood flow in ferric chloride experiment

Percent blood flow was calculated with the help of LDF reading obtained before and after the occlusion of the carotid artery [32].

Percentage blood flow obstruction was calculated by using the following formula:

$$Percentage\;Blood\;flow\;obstruction\;(\%)=\frac{Normal\;LDF\;reading-\;After\;occlusion\;LDF\;reading}{Normal\;LDF\;reading}\;X\;100$$

PercentageBloodflow(%)=100-%Bloodflowobstruction

#### Histopathology of carotid artery

To further confirm the formation of thrombus in the carotid artery, mice (n = 6) were monitored for one hour post ferric chloride exposure and thereafter euthanized using an overdose of cocktail injection of ketamine and xylazine. One mm of proximal and one mm of distal normal artery along with the injured arterial segments were excised (total length of the arterial segment, 4–5 mm), placed in 10% buffered formalin solution. Paraffin-embedded sections of4-5 μm thickness were cut and stained with hematoxylin and eosin (H&E) staining.

#### Fluorescence molecular tomographic imaging of mice

Fluorescence molecular tomographic (FMT) imaging of mice was performed with the FMT2500 Lx (Perkin Elmer Life Sciences, Waltham, MA). 24 hours prior to imaging, AngioSense 750x was injected intravenously (IV) in mice (n = 3) of three different groups. After 24 hours, mice were pretreated either with vehicle or rhuGSN (8 mg/mouse, SC) about 30 minutes prior to the application of ferric chloride solution. In the normal control group, the carotid artery was not subjected to ferric chloride injury. All the procedures on mice were performed under isoflurane anaesthesia. Thrombosis was induced in the carotid artery using ferric chloride as described previously[33]. Mice were then imaged to assess the injury to carotid endothelium following ferric chloride exposure. TrueQuant software was used for processing and analysis of images.

### Thrombin-induced acute pulmonary thromboembolism

We investigated the effect of rhuGSN in the thrombin-induced acute pulmonary thrombosis mouse model as described earlier [34]. Mice (n = 6) were divided into different groups; *viz*. control, vehicle, aspirin, rhuGSN and fasted overnight before the experiment. Half an hour prior to the injury, Aspirin (75mg/kg, intraperitoneal, IP) and rhuGSN (8mg/mouse, SC) were administered. To induce thrombosis, thrombin (20 U) was injected in the tail vein of mice in all groups except control. Mice were observed for mortality and paralysis for 15 minutes and lung sections of different groups of mice were collected for H&E staining after euthanasia.

Percent protection in each group of mice was calculated by using the following formula[35].

$$Percent\;protection\;(\%)=(1-\frac{Dead\;or\;paralyzed\;mice}{Total\;mice})X\;100$$

#### Bleeding time assay and whole blood platelet count

In another experiment, standard protocol was followed for amputating 3 mm tail slice from the tip of the tail of mice treated with vehicle and rhuGSN (n = 6). Tissue paper was used to remove unclotted blood at incision site after every 15 seconds. The period from the beginning to the cessation of bleeding was recorded as the bleeding time[36, 37]. In a separate protocol, mice (n = 6) pretreated either with aspirin or rhuGSN were subjected to the vehicle or thrombin-induced injury and studied in a similar way. Blood samples from these mice were collected and platelets were counted using Haematological Auto-analyser.

### Statistical analysis

All data represent the median value ± SD. The Chi-square test was applied to compare mortality data in different groups of mice. One-way analysis of variance (ANOVA), followed by Dunnet’s test for multiple comparisons was used for other studies using GraphPadInStat 3. Test results were considered significant when p<0.05.

## Results

### Ferric chloride-induced arterial thrombosis

**Fig 1:**
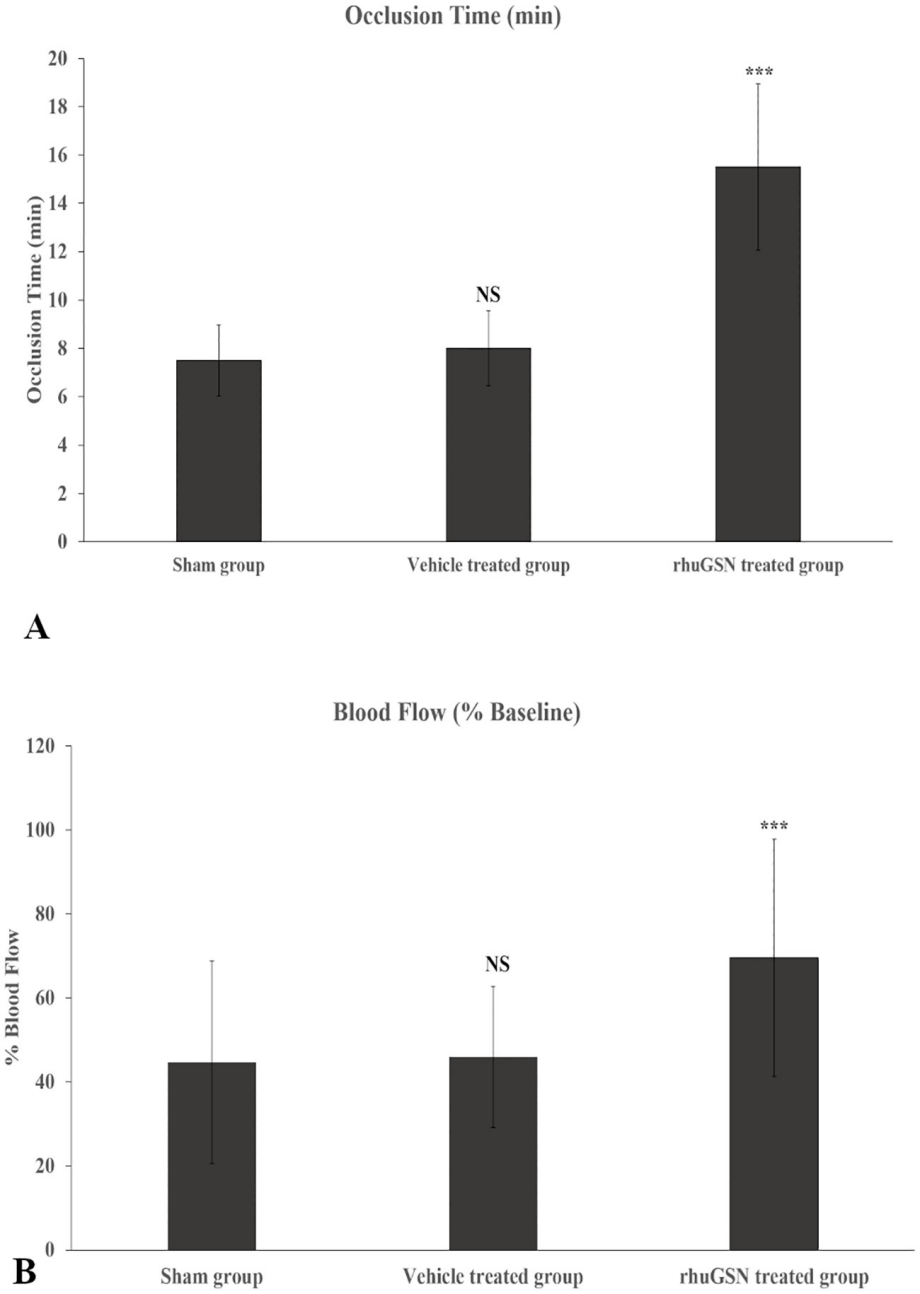
Effect of rhuGSN in ferric chloride induced thrombosis in mice on (A) Occlusion time and (B) Blood flow. Administration of rhuGSN (8 mg/mouse) prolonged the occlusion time and increased blood flow after arterial injury. Data are presented as the median ± SD (n = 6). ***P <0.001, *P <0.05. NS: not significant.

Application of ferric chloride on the surface of the carotid artery severely damages the endothelial lining due to oxidative stress and causes occlusion of the artery through platelet-rich thrombi[38]. To evaluate the role of GRT in rescuing mice from thrombosis of the carotid artery, blood flow was monitored using LDF. Time for occlusion of blood vessel in injury-induced and vehicle-treated mice was about 8 minutes. Notably, this occlusion time was almost double i.e. 15.50 minutes in mice treated with rhuGSN (Fig 1A). It is pertinent to mention here that the application of filter paper alone (i.e. no ferric chloride) to carotid arteries did not induce thrombosis. We also measured blood flow in the carotid artery of normal (sham group) as well as ferric chloride- exposed mice pretreated either with the vehicle or rhuGSN. Percent blood flow in the carotid artery following ferric chloride injury was increased by approximately two-fold in mice pretreated with rhuGSN (Fig 1B).

#### Histology of ferric chloride induced thrombosis of carotid artery

**Fig 2:**
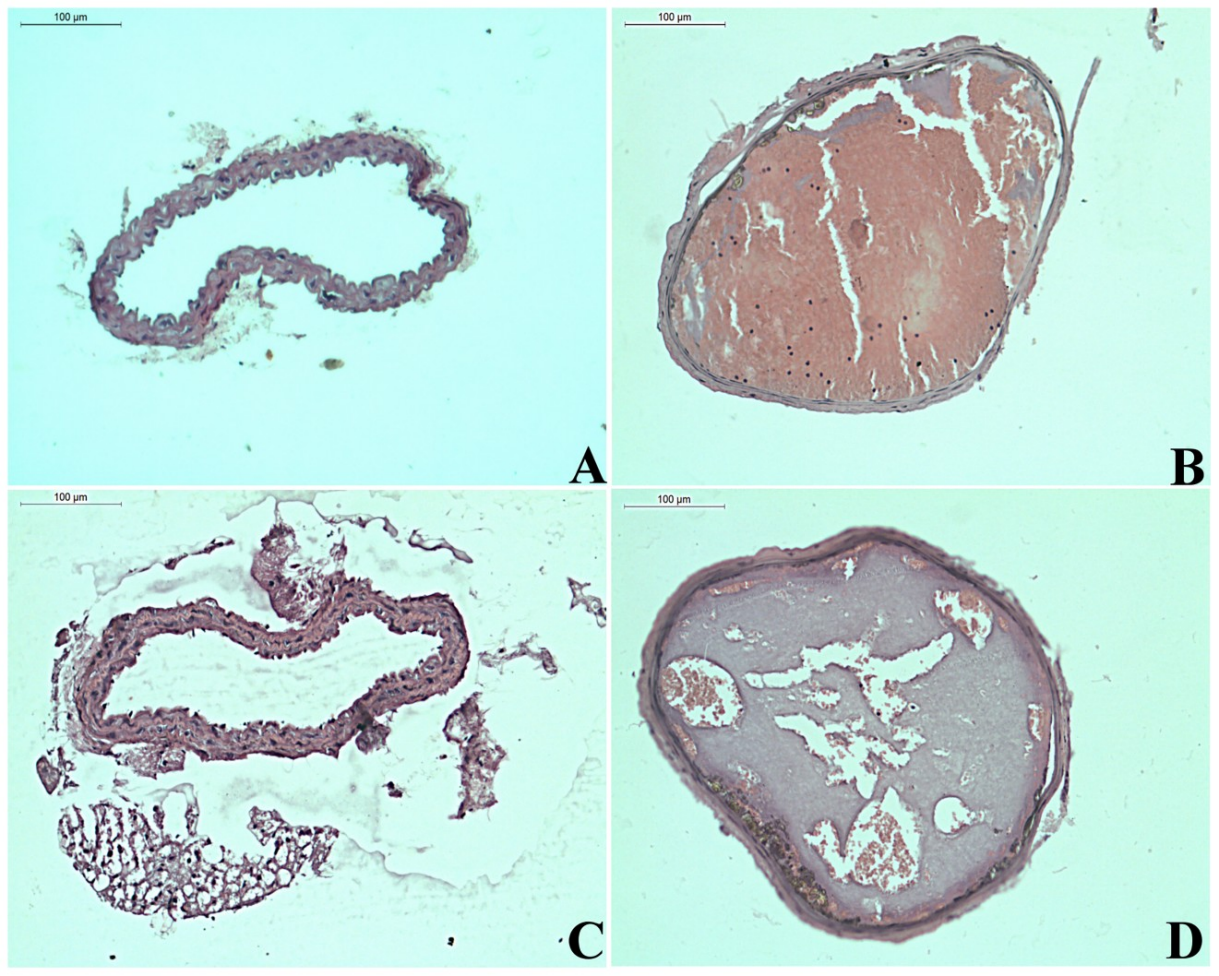
Representative micrographs of H&E-stained carotid artery (A) Normal (sham) carotid artery (B-D) Carotid artery following ferric chloride injury. Section of (B) Vehicle treated group, (C) Heparin (20U/kg) treated group and (D) rhuGSN (8mg/mouse) treated group (Scale bar = 100μm). In rhuGSN treated mice, significantly reduced damage to endothelial and substantially reduced aggregation of platelets was observed.

As expected, histological sections of mice in the sham group depicted normal endothelial surface, however, injury-induced vehicle treated group showed occlusion in the lumen of the vessel (platelet thrombi) and damaged endothelium with reduced thickness[39]. Expectedly, injury-induced heparin treated mice exhibited continuous endothelium throughout the luminal surface in the carotid artery. Interestingly, administration of a single dose of rhuGSN to mice prior to injury provided significant protection to endothelium and reduced aggregations of platelets in the arterial lumen (Fig 2).

#### Fluorescence molecular tomographic imaging of mice

**Fig 3:**
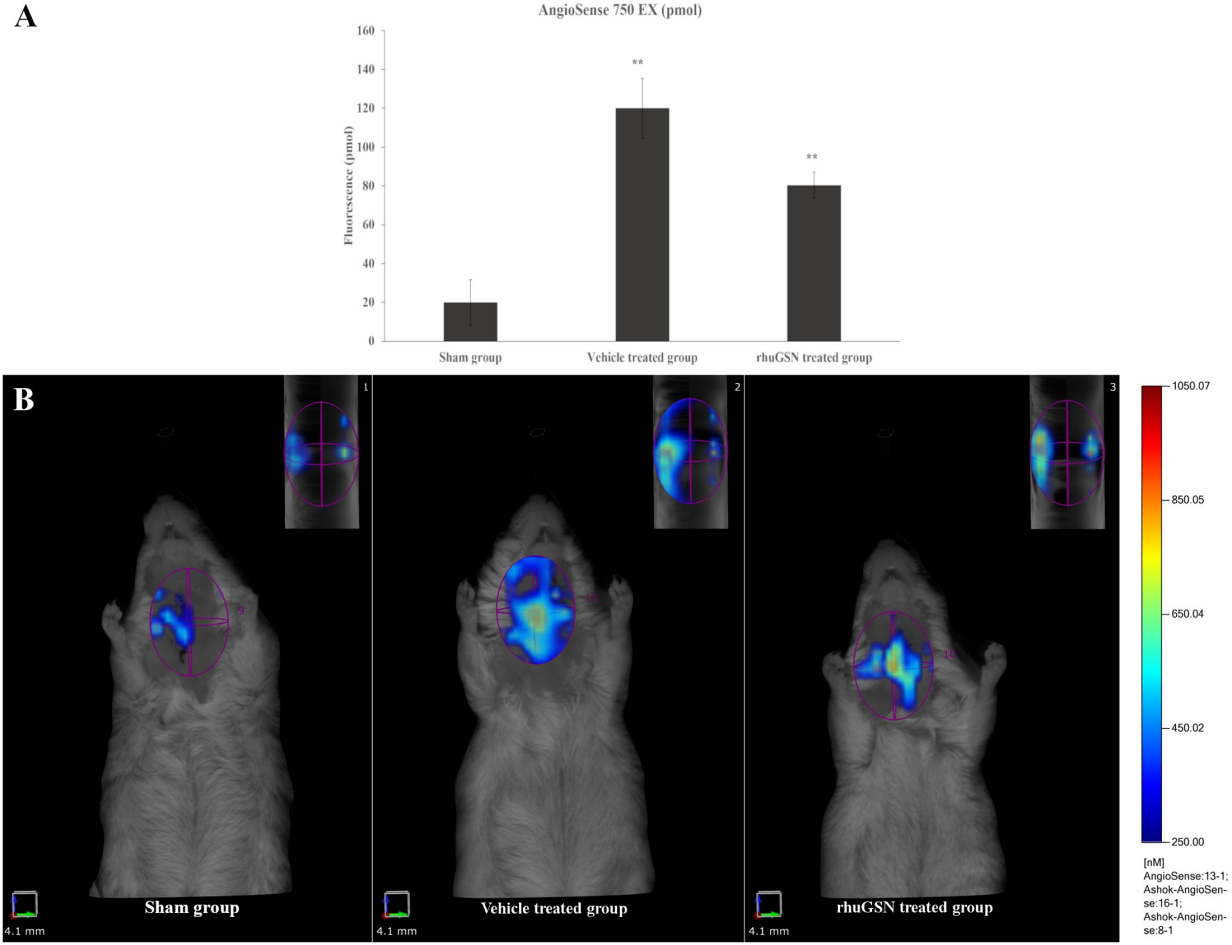
Fluorescent tomographic imaging of mice (A) Bar diagram representing the quantification of fluorescence (Thrombosis) (B) Fluorescent tomographic imaging of carotid artery (n = 3) probed with AngioSense 750 EX showing a reduction in arterial injury upon pretreatment with rhuGSN. Images in inset represent 90° view of corresponding 3D geometry of the carotid artery in mice of different groups. Data are presented as the median± SD (n = 3). **P <0.01.

Ferric chloride- induced thrombosis group of mice depicted a severe injury to the carotid artery as evident by a six-fold increase in the fluorescence intensity as compared to the normal (sham)group. Significant protection (40%) was observed in injury-induced group pretreated with a single dose of rhuGSN. The reduction in intensity of fluorescence in the rhuGSN treated group indicates the protective role of rhuGSN in preventing thrombosis (Fig 3A & 3B).

### Effect of rhuGSN on thrombin-induced acute pulmonary thromboembolism in mice

**Fig 4:**
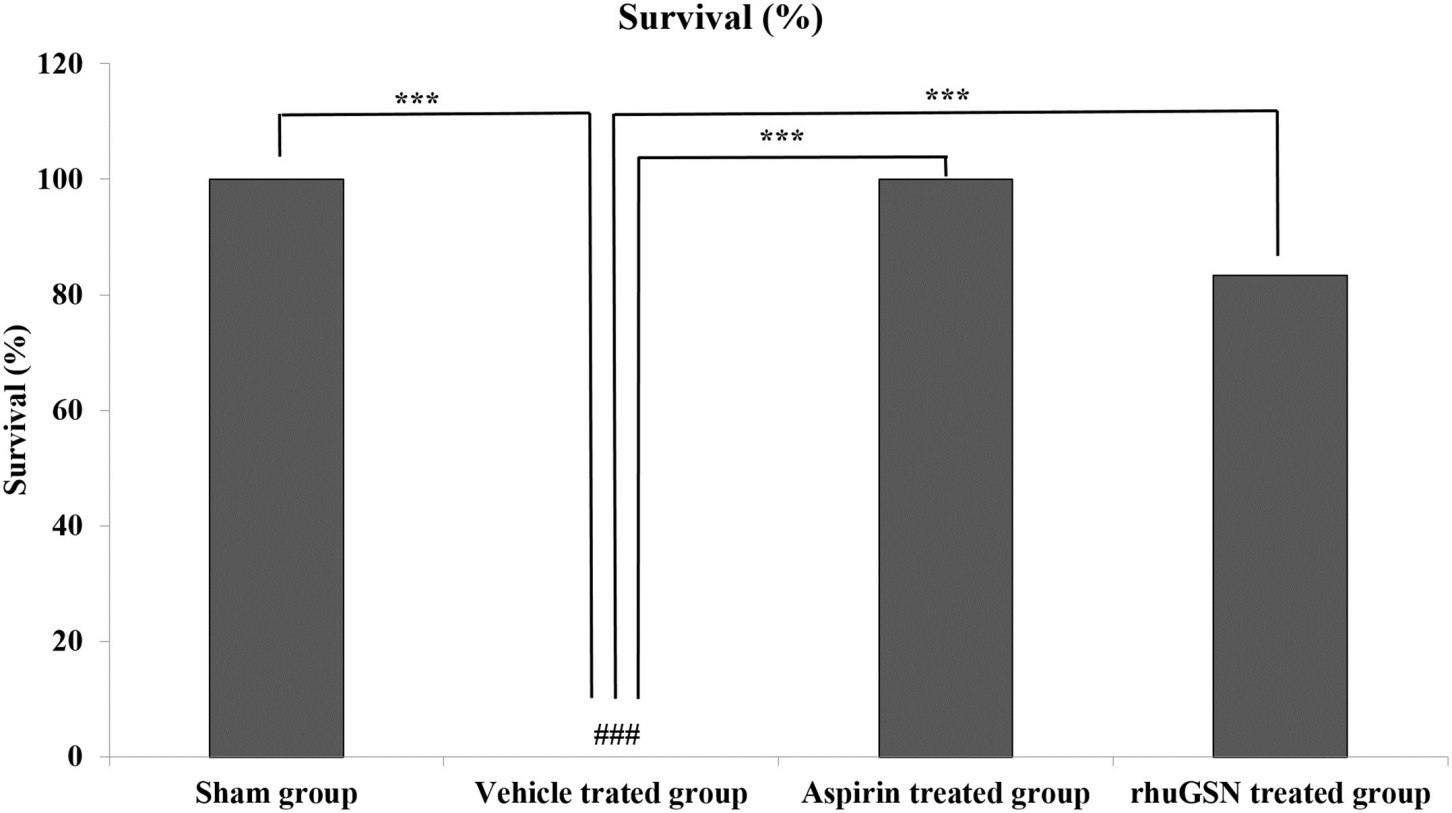
rhuGSN pretreated mice were protected from thrombin-induced thromboembolic mortality. Mice (n = 6) were pretreated with vehicle, 8 mg/mouse rhuGSN or 75 mg/kg aspirin 30 minutes prior to intravenous injection of 20 IU thrombin. GRT was effective in preventing thrombin-induced thromboembolic death. Data are presented as the median± SD. ***P <0.001.

Thrombin-induced acute pulmonary thromboembolism in mice was induced as previously described[40]. In this model, thrombin injection in the tail vein of mice caused platelet activation that led to the formation of thrombi in lungs and resulted in complete mortality in the vehicle treated group, whereas mice treated with aspirin showed 100% survival. Intriguingly, rhuGSN showed significant antithrombotic activity and 83.33% of mice survived the thrombin challenge (Fig 4).

#### Bleeding time assay and platelet count in mice

**Fig 5:**
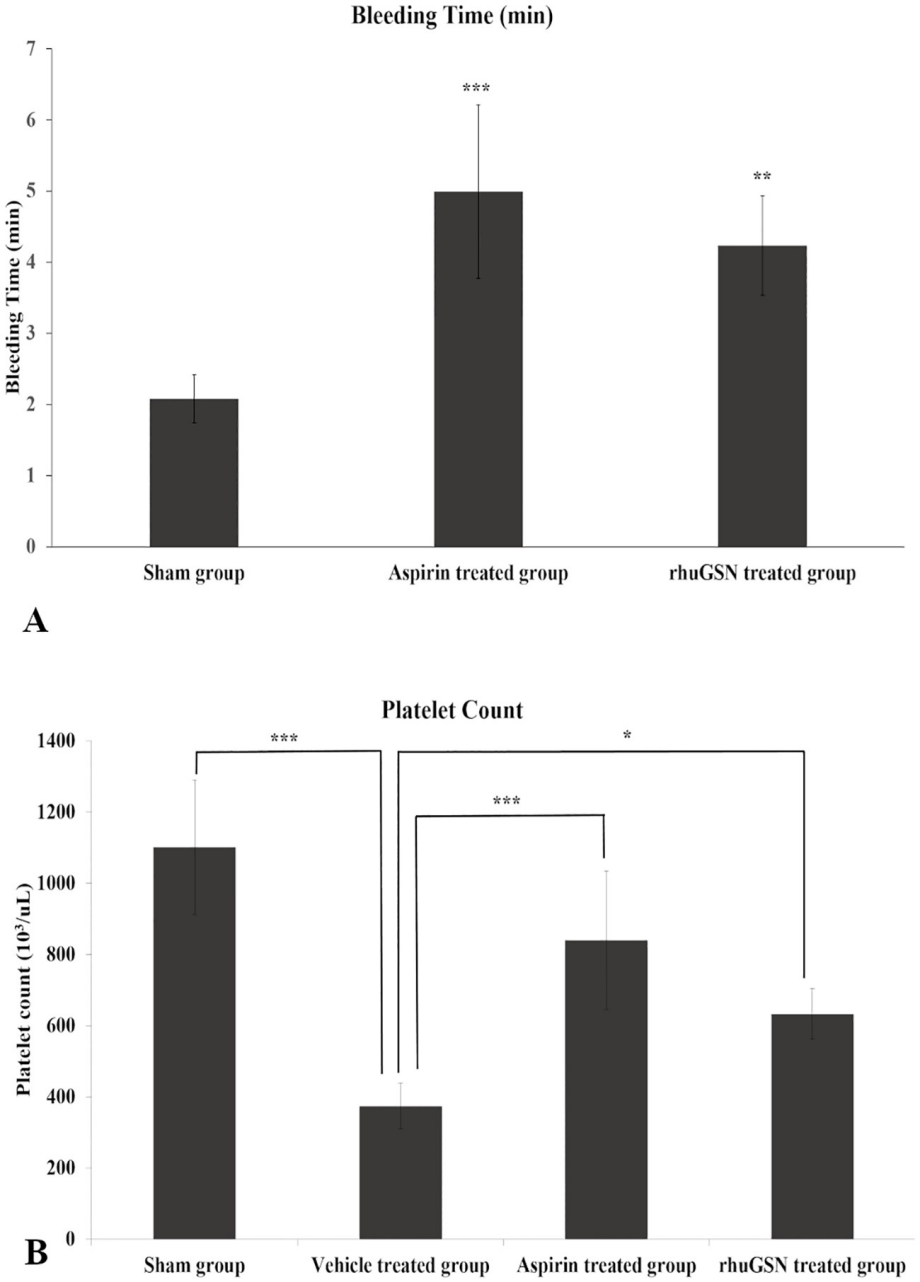
Effect of rhuGSN on (A) Bleeding time and (B) Platelet count in thrombin-induced acute pulmonary thromboembolism. Data are presented as the median± SD (n = 6). *P <0.05, **P <0.01, ***P <0.001.

We investigated the role of rhuGSN in modulating the bleeding time and platelet count. As shown in Fig 5A, mice treated with aspirin and rhuGSN showed an approximately two-fold increase in bleeding time than vehicle treated mice. Thrombin treated mice (388.00 x 10^3^/ml) showed a considerable decline in platelet numbers as compared to normal (sham) mice (1142.83 x 10^3^/ml). On the other hand, platelet count in mice pretreated with aspirin and rhuGSN were 851.33 x 10^3^/ml and 624.17 x 10^3^/ml, respectively. It can be estimated that in the time window of experimentation, thrombin reduced platelet count by 70% whereas pretreatment with aspirin and rhuGSN led to a reduction in platelet count by only 25 and 50%, respectively. These results suggest that rhuGSN could significantly protect mice from thrombin-induced acute pulmonary thromboembolism (Fig 5B).

#### Histopathological examination of lungs

**Fig 6:**
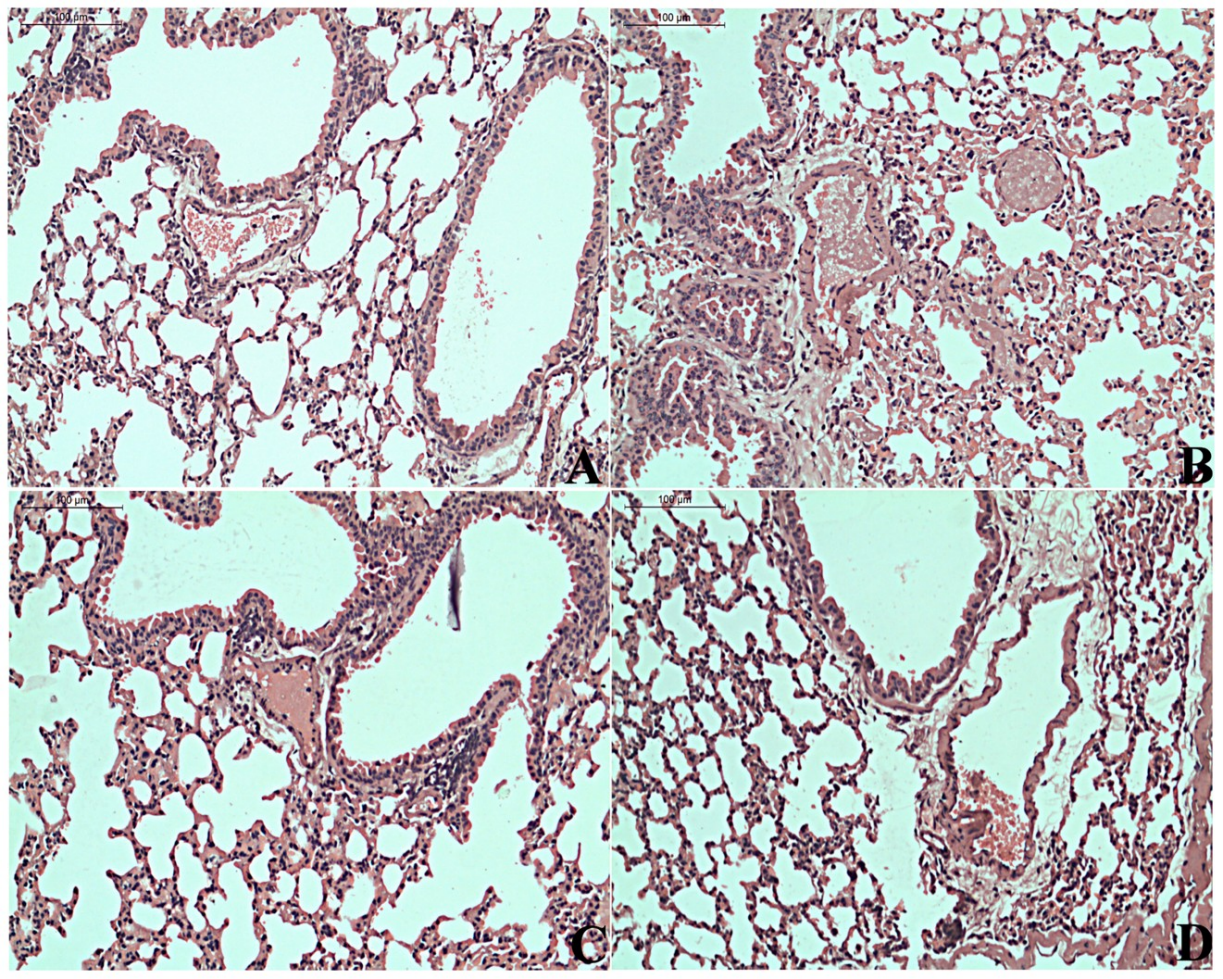
rhuGSN- or aspirin-treated mice were protected from microvascular thrombosis. Mice were pretreated with vehicle, 8 mg/mouse rhuGSN or 75 mg/kg aspirin 30 min prior to injury with intravenous injection of 20 IU thrombin. Representative H&E stained images of the histological section of (A) normal lung (sham group), (B-D) thrombin-treated. Sections of (B) vehicle treated group, (C) aspirin-treated and (D) rhuGSN treated group, (Scale Bar = 100μm).

Lung tissues were fixed in 10% buffered formalin and stained with hematoxylin-eosin stain to study the effect of rhuGSN on thrombin-induced pulmonary thromboembolism. Platelet thrombi were observed in lung sections of thrombin and vehicle treated mice. On the other hand, treatment of mice either with 8 mg/mouse rhuGSN or 75 mg/kg aspirin resulted in a significant reduction in platelet thrombi in the lung parenchyma (Fig 6). This data explained the observed protection in mortality in mice injected with thrombin that is partly due to modulation of bleeding time, platelet count and reduced platelet thrombi in rhuGSN pretreated mice.

## Discussion

With the increase in sedentary lifestyle, uncontrolled calorie and imbalanced nutritional intakes, metabolic disorders particularly cardiovascular, cerebrovascular diseases and venous thromboembolism are emerging as major pathological disorders responsible for high mortality in humans, thus significantly increasing the healthcare costs[1, 2]. Current treatment options for thrombosis are not adequate, therefore, there is a need for new biomolecules which can provide a long-lasting and holistic antithrombotic activity. Minimalistic approaches such as protein-analysis or cell-line based experiments provide useful information but can not substitute animal experimentation where it is important to understand the complete balance between biochemistry to tissue. Thus, we opted to perform animal experiments that provide a complete mapping of the disorder and intervention profile. As introduced, decreased gelsolin levels have been reported in many human diseases as well as in animal models. GRT is showing promising rescue in many conditions and thus is under clinical trials for different critical care indications. More relevant to our work, administration of recombinant gelsolin has been shown to have a protective effect in murine stroke[22]. It correlates with long-term tracking of response and quality-adjusted life of stroke patients with their plasma gelsolin levels[27]. Interconnecting the therapeutic promise of GRT, and its potential role in maintaining cardiovascular balance, we carried out animal studies to examine the antithrombotic activity of gelsolin.

Both, ferric chloride induced thrombosis in the carotid artery and thrombin-induced pulmonary thromboembolism models are well-established for these indications and have been used extensively for *in vivo* testing of new antithrombotic therapies[41, 42]. Ferric chloride induced thrombosis is the model of choice as factors such as the site of arterial injury, the duration of injury and the concentration of ferric chloride can be precisely controlled, hence, uniform injury can be induced every time. Additionally, the blood flow rate in the carotid artery can be monitored accurately in mice using various blood flow meters. It is well established that the application of ferric chloride causes free radical induced oxidative stress, majorly by lipid peroxidation[39]. Ferric chloride diffuses through the vessel wall, causing the destruction of endothelial wall and formation of ferric-ion filled spherical bodies, which in turn induce platelet activation, adhesion and thrombus formation[43]. Platelet thrombi formation occludes blood flow rate which is a quantitative measure of vasculature damage. Thus, this model pathologically resembles a thrombolytic disease[44]. In our experiments, we found that a single dose of 8 mg of rhuGSN per mice increased both blood flow in the carotid artery and the time for occlusion of an artery following ferric chloride injury. FMT based imaging of injured artery and histopathological examination of arterial sections further confirmed the protective effect of rhuGSN. These results are consistent with the earlier findings wherein gelsolin was reported to exert antioxidant property by decreasing lipid peroxidation and increasing levels of glutathione (GSH) in radiation induced injury to mice[45] and acute oxidative lung injury[18].

In thrombin-induced acute pulmonary thromboembolism, as compared to complete mortality in vehicle treated group of mice within 15 minutes of challenge, rhuGSN increased survival rate and reduced thrombus formation. In addition, intravenous injection of thrombin to mice led to a severe decline in platelet count. Conversely, platelet count did not decrease in aspirin and rhuGSN pretreated mice. Histopathological examination of lung tissue showed that thrombin infusion caused widespread platelet thrombi within the lung vasculature. Treatment of rhuGSN reduced the formation of thrombi as well as mortality in mice. Administration of aspirin and rhuGSN substantially extended the bleeding time as compared to vehicle treated mice. Platelets play a central role in arterial thrombosis; hence, anti-platelet therapy is advantageous in prevention of thromboembolic diseases. Haddad *et*.*al*. demonstrated that the formation of pulmonary thrombi in rats was due to aggregation of platelets upon intravenous injection of G-actin. Treatment of G-actin with Vitamin D binding protein could avert thrombus formation[46]. Although the role of gelsolin as actin regulatory protein has been well established, the direct role of gelsolin in thrombus formation has not been examined [47, 48].

It has been reported that large quantities of actin are released from injured cells into the extracellular space following tissue damage[47]. Because gelsolin is involved in severing and capping of actin filaments, therefore, a high amount of gelsolin is depleted and utilized to remove actin from the circulation. This hypothesis is correlated with the fact that exhausted levels of gelsolin had previously been reported in many disease conditions and injuries[17–23, 28, 29, 49]. F-actin is directly responsible for aggregation of platelets *in vitro* and increased amount of F-actin in circulation may lead to clogging of micro vessels, thereby reducing the flow of blood and generation of thrombus in the blood vessels[44, 50]. Huang *et al*. proposed that actin filaments have a direct or indirect role on activation and aggregation of platelets through direct injury to endothelium or by the release of mediators such as thromboxane and lysophosphatidic acid[51]. As gelsolin is actin regulatory protein and has been shown to bind to lysophosphatidic acid[52]; it can have an indirect effect on platelet aggregation by scavenging actin from the circulation and restoring the blood flow in the injured carotid artery. Actin interacts with platelets, plasmin, and fibrin following cell injury and integrates into fibrin to form a coarse clot[53]. Gelsolin participates in clot lysis by preventing the incorporation of actin into the fibrin clot and thereby impedes the formation of coarse fibrin mass. In addition, gelsolin breaks actin filaments trapped in a fibrin clot into shorter fractions and leads to removal of actin from the clot thus making it more susceptible to lysis [53, 54]. As per literature, our results provide first direct evidence on the antithrombotic activity of rhuGSN in ferric chloride-induced carotid arterial injury and protection of mice in acute pulmonary thromboembolism. The precise mechanism by which rhuGSN achieves the observed activity will remain part of our future research activity. Very likely, calcium-activated plasma gelsolin rapidly depolymerizes F-actin released in blood or blocks rapid polymerization of G-actin released in high ionic strength of blood. Either way, gelsolin lowers blood viscosity and possibly enables availability of other factors essential for repair.

Although results reported in this study are exciting, there are a few limitations of this study and GRT for patients with thrombotic issues, i. Control experiments will be required for dose optimization in correlation with variances in the extent and order of damage to the thrombotic system, ii. rhuGSN is not commercially available to conduct trials in phases. Solinex^™^, the injectable form of rhuGSN has been discontinued, iii. The inherent ability of full-length gelsolin to tightly bind to LPS and LTA, which has limited use of regular bacteria for recombinant protein production. In this aspect, its thermostable bonsai versions that completely lack binding to LPS or LTA but can depolymerize F-actin faster than parent molecule can be considered as therapeutic alternatives or complements to gelsolin. Bearing all these challenges in the total translation of our work to human clinical trials, the study is a promising start and we believe our work would inspire further work in this direction.

## Conclusion

We for the first time report the thrombo-protective capability of exogenous gelsolin in ferric chloride-induced thrombosis of carotid artery and thrombin induced acute pulmonary thromboembolism in mice. The mechanism involved in this pGSN–mediated protection, however, requires further investigation, but it opens up the possibility of GRT as a thrombo-protective option.
